# Plasma Total Cysteine and Cardiovascular Risk Burden: Action and Interaction

**DOI:** 10.1100/2012/303654

**Published:** 2012-04-19

**Authors:** Benedetta De Chiara, Valentina Sedda, Marina Parolini, Jonica Campolo, Renata De Maria, Raffaele Caruso, Gianluigi Pizzi, Olga Disoteo, Cinzia Dellanoce, Anna Rosa Corno, Giuliana Cighetti, Oberdan Parodi

**Affiliations:** ^1^CNR Clinical Physiology Institute and Cardiovascular Department, Niguarda Ca' Granda Hospital, 20162 Milan, Italy; ^2^Diabetologic and Metabolic Unit, Niguarda Ca' Granda Hospital, 20162 Milan, Italy; ^3^Department of Clinical Sciences “Luigi Sacco”, University of Milan, 20157 Milan, Italy

## Abstract

We hypothesized that redox analysis could provide sensitive markers of the oxidative pathway associated to the presence of an increasing number of cardiovascular risk factors (RFs), independently of type. We classified 304 subjects without cardiovascular disease into 4 groups according to the total number of RFs (smoking, hypertension, hypercholesterolaemia, hyperhomocysteinaemia, diabetes, obesity, and their combination). Oxidative stress was evaluated by measuring plasma total and reduced homocysteine, cysteine (Cys), glutathione, cysteinylglycine, blood reduced glutathione, and malondialdehyde. Twenty-seven percent of subjects were in group 0 RF, 26% in 1 RF, 31% in 2 RF, and 16% in ≥3 RF. By multivariable ordinal regression analysis, plasma total Cys was associated to a higher number of RF (OR = 1.068; 95% CI = 1.027–1.110, *P* = 0.002). Total RF burden is associated with increased total Cys levels. These findings support a prooxidant effect of Cys in conjunction with RF burden, and shed light on the pathophysiologic role of redox state unbalance in preclinical atherosclerosis.

## 1. Introduction

Cardiovascular risk factors (RFs), such as smoking, hypertension, hypercholesterolaemia, diabetes mellitus, and hyperhomocysteinaemia, are closely related to the development of atherosclerosis [1]. Several experimental and clinical studies support the notion that oxidative stress plays a significant role in this process [2].

Hyperhomocysteinaemia is a nonclassical RF independently associated to the development of cardiovascular disease [3]. The mechanism by which homocysteine promotes the production of hydroxyl radicals and lipid peroxidation initiators may be related to the reactivity of the sulfhydryl group leading to homocysteine autooxidation and thiolactone formation. This is of clinical relevance, because mild to moderate hyperhomocysteinaemia, even though not atherogenic* per se*, may lead to progression of atherosclerosis in concert with other classical RFs.

Other thiols might also be expected to confer increased cardiovascular risk. Cysteine (Cys) exhibits chemical properties similar to homocysteine through its sulfhydryl (–SH) group, and its levels in plasma are 20-fold higher than homocysteine [4]. However, its potential noxious effects in cardiovascular disease have till now received little attention [5–7].

Homocysteine and Cys react via redox pathways in the extracellular space, and their reduced, oxidized, and protein-bound forms participate in the dynamic system known as redox thiol state. Since thiol/disulfide redox states, *per se*, are crucial both in redox signalling and control and in antioxidant protection, they play an important role in the connection between environmental influences and progression of detrimental changes associated with the presence of RF.

Epidemiological research documented an independent U-shaped relationship between total Cys and overt cardiovascular disease, even after adjustment for homocysteine, creatinine, and other cardiovascular RFs [7]. Indeed, another study reported that, among all thiols, the steady redox-state of plasma Cys is the most oxidized one [8].

The aim of the present study was to investigate in a population with no overt cardiovascular disease the relationship between the cardiovascular RF burden and redox state and lipid peroxidation, in terms of aminothiol and free malondialdehyde (MDA) concentrations.

## 2. Materials and Methods

### 2.1. Study Population

We prospectively enrolled 304 subjects (169 males, aged 53 (43–63) years) selected among 793 consecutive subjects, who had been referred to our centre for evaluation of the redox pattern. Patients presenting the following were excluded from the study: moderate to severe kidney dysfunction (serum creatinine ≥2 mg/dL), uncontrolled hypertension (blood pressure levels ≥180/110 mmHg), congestive heart failure, unstable angina, previous myocardial infarction, angiographically documented coronary artery disease, ischemic stroke, known neoplasms, ongoing infection, immune system disease, type 1 or type 2 diabetes mellitus with micro- or macrovascular complications, advanced liver disease, and alcohol abuse meeting the criteria of DSM-III-R American Psychiatric Association [9].

No subject had been treated with antioxidant vitamins supplement, *N*-acetylcysteine, or Cys supplements within 2 months before enrolment.

Written informed consent for study participation was obtained from all patient. The study protocol was approved by the Niguarda Hospital Ethics Committee.

### 2.2. RF Assessment

Details on commonly implicated RFs and general dietary and medical history and lifestyle information were obtained from each subject using a questionnaire. Assessed RF were smoking, hypertension, hypercholesterolaemia, hyperhomocysteinaemia, uncomplicated type 2 diabetes, and obesity. Subjects were considered smokers if they had smoked at least 10 cigarettes per day in the previous year, with no interruption longer than 3 months [10]. Hypertension was defined as systolic blood pressure >140 mmHg and/or diastolic blood pressure >90 mmHg on repeated measurements or on current treatment with antihypertensive agents; lower blood pressure cutoff values (systolic blood pressure >130 mmHg and/or diastolic blood pressure >80 mmHg) were used to identify diabetic subjects as hypertensive [11]. Hypercholesterolaemia was defined as low-density lipoprotein (LDL) cholesterol level ≥160 mg/dL or current treatment with lipid-lowering medications [12]. Hyperhomocysteinaemia was defined as total plasma homocysteine ≥15 *μ*mol/L [13]. Type 2 diabetes mellitus was defined using established criteria [14] or by being on current treatment with hypoglycaemic agents. Obesity was defined as body mass index ≥30 kg/m^2^ [15].

We assessed the clustering of RFs (smoking, hypertension, hypercholesterolaemia, hyperhomocysteinaemia, diabetes, and obesity) for each subject, with a range of 0 (none of these conditions) to ≥3 (three or more conditions).

### 2.3. Blood Sample Collection

Peripheral venous blood was sampled in the morning after overnight fasting in the sitting position. Blood samples were collected in the Vacutainer tubes and immediately processed for blood, plasma, and serum determinations.

### 2.4. Redox State Determination

The endogenous redox state, defined as reduced and oxidized aminothiol equilibrium, was evaluated by measuring blood and plasma concentration of reduced and total homocysteine, Cys, glutathione, and cysteinylglycine. For total aminothiols, an aliquot of whole blood was diluted with an equal volume of distilled water and frozen in liquid nitrogen; the remaining blood was centrifuged (2000 g for 10 min at 4°C) within 10 minutes of collection to obtain plasma, which was aliquoted, frozen, then stored at −80°C, and analyzed within 1 week. The total forms measured in our laboratory include the oxidized aminothiols (disulfides), all conjugated forms (among them protein-bound aminothiols and mixed aminothiols-disulfides produced through oxidative processes or thiol-disulfide exchange reactions), and reduced free aminothiols. Blood and plasma reduced aminothiols were determined by prompt acidification with 10% trichloroacetic acid (1 : 1, v/v), protein precipitation, and sample derivatization with ammonium-7-fluorobenzo-2-oxa-1,3-diazole-4-sulphonate, a specific reagent for –SH groups. Plasma total aminothiols were instead measured after a reducing step with tri-*n*-butylphosphine, followed by sample derivatization with the same agent described below. Thiol concentrations were determined by isocratic high-performance liquid chromatography (HPLC; Varian, Surrey, UK) on a Discovery C18 column (250 × 4.6 mm I.D, Supelco, Sigma-Aldrich) and eluted with a solution of 0.1 mol/L potassium dihydrogenphosphate-acetonitrile (92 : 8, v/v), pH 2.1, at a flow rate of 1 mL/min, as previously described [16–18]. Fluorescence intensities were measured with excitation *λ* at 385 nm and emission *λ* at 515 nm, using a JASCO fluorescence spectrophotometer.

### 2.5. Lipid Peroxidation Assessment

Levels of free MDA, the reactive nonconjugated MDA form, were assessed in stored plasma using selected ion-monitoring gas chromatography-mass spectrometry (GC-MS, Hewlett-Packard Company, Palo Alto, Calif, USA) in electron impact mode, equipped with an HP-5 fused silica capillary column (25 m, 0.32 mm I.D., 0.25 *μ*m film thickness). Plasma samples were spiked with dideuterated internal standard and derivatized with phenylhydrazine hydrochloride before GC-MS analysis. The UV determinations were carried out with a Perkin-Elmer Lamda-11 spectrophotometer [19]. Values are expressed in *μ*mol/L.

### 2.6. Other Chemical Evaluations

Vitamin B_12_ and folates were measured by competitive immunoassay using direct chemiluminescence, while glucose, creatinine, fibrinogen, total cholesterol, and triglycerides were determined using standard laboratory methods. High-density lipoprotein (HDL) cholesterol was measured after precipitation with dextran sulfate-magnesium and LDL cholesterol was calculated using Friedewald's method.

### 2.7. Statistical Analysis

Discrete variables are presented as frequency percent. Continuous variables are expressed as median and interquartile range [I; III]. Univariable ordinal logistic regression analysis was used to study the association between the cumulative number of RFs and clinical, biochemical, and redox state variables, adjusting for age and gender. Results are presented as odds ratio (OR) and their 95% confidence interval (CI). Significant variables (*P* < 0.1) were entered in the final multivariable model.

The statistical analyses were carried out with the Statistical Package for the Social Sciences (SPSS Inc., Chicago, Ill, USA), Release 10.0 for Windows.

## 3. Results

Cardiovascular RF distribution in the study population was as follows: smoking (*n* = 86, 28%), hypertension (*n* = 102, 33%), hypercholesterolaemia (*n* = 77, 25%), diabetes (*n* = 50, 16%), hyperhomocysteinaemia (*n* = 72, 24%), and obesity (*n* = 34, 11%).

Study subjects were categorized into 4 groups based on the number of cardiovascular RFs (group 0, 1, 2, and 3 if ≥3 RF). The control group included 81 (group 0, 27%) subjects, while 95 (group 1, 31%) had 1 RF, 80 (group 2, 26%) had 2, and 48 (group 3, 16%) had 3 or more RFs.

We performed an analysis on the clustering of RF, irrespective of type. The clinical and biochemical characteristics of the RF groups are described in Table 1, and the redox state profile is described in Table 2. Patients with more RF were older and more frequently males and had higher levels of fasting glucose, total cholesterol, and triglycerides than those with less or no RFs. As regards the redox pattern, plasma total Cys and plasma free MDA levels increased with RF number, although without achieving statistical significance, while the other variables did not show any marked linear trend.

Current drug treatment of study subjects is shown in Table 3. No subject in group 0 took medications. As expected, consistently with current guideline recommendations, the proportion of subjects who took drugs for the treatment of specific RF, in most instances with drug classes with recognized antioxidant properties, increased with increasing RF number.

**Figure 1:**
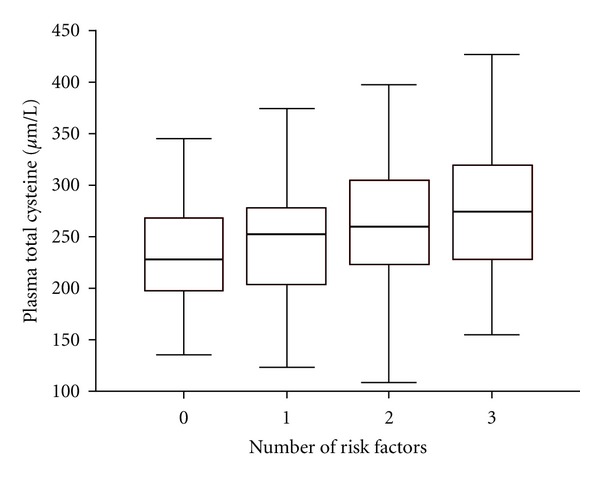
Plasma total cysteine and risk factors burden in study subjects. Raised plasma total cysteine concentrations are independently associated with the presence of a higher number of risk factors. The box plots (*μ*mol/L) show values expressed as median (50th percentile), interquartile ranges (25th and 75th percentiles, box), and extreme values (whiskers) of total plasma cysteine in relation to risk factor grouping in subjects without (0) and with (1, 2, and 3) cardiovascular risk factors.

The association between biochemical and redox state variables and the RF burden was tested by univariable ordinal logistic regression analysis, with age and gender as covariates, to adjust for the observed unbalance in study groups. All the variables that reached the significance level *P* < 0.10 (Table 4) were entered into the final multivariable ordinal regression model. The analysis revealed that plasma total Cys (OR = 1.068; 95% CI = 1.027–1.110, *P* = 0.002) was the sole redox state variable independently associated with the number of RFs. Distribution of plasma total Cys levels according to RF burden is shown in Figure 1. The only other independent predictor of RF burden was the number of administered medications (OR = 3.710; 95% CI = 2.680–5.136, *P* < 0.001).

## 4. Discussion

In the present study, we analyzed in a large number of subjects without overt cardiovascular disease the association between the total RF burden and the redox state. Among all redox variables, total Cys was the only parameter significantly associated to RF burden.

Many cardiovascular RFs physiologically interact in the aetiology of cardiovascular disease. In addition, clustering of RFs is frequent and may increase the risk multiplicatively [20].

The potential vascular toxicity of Cys has recently been emphasized, though this aminothiol has not been as widely studied as homocysteine. In vitro, autooxidation of Cys occurs more readily than that homocysteine, thereby generating reactive oxygen species that can, by oxidation, modify low-density lipoproteins [21]. Additionally, Cys effects endothelium-dependent contraction by generating O_2_^·−^, which rapidly inactivates the endothelium-derived relaxing factor [22]. In the experimental setting, auto-oxidation of homocysteine was found to be dramatically accelerated by the presence of either Cys or cystine [23]. Cys, and not homocysteine, has demonstrated a high reactivity towards DNA, both in the absence and presence of copper(II), leading to the formation of reactive species and the induction of oxidative stress [24]. Finally, Cys more readily undergoes autooxidation at physiologic pH, because of its lower pK values than all other thiols [21].

In our series, plasma reduced Cys showed no consistent trend in relation with the number of RF. Therefore, the direct association of plasma total Cys, which includes free reduced, free oxidized, and protein-bound forms, with a greater RF burden may be ascribed mainly to increased Cys oxidation processes. These covalent modifications favour an enhanced oxidative alteration, which leads to changes in protein structure and function.

Our results are in agreement with previous reports of increased total Cys levels in patients with vascular diseases [7]. However, those studies were performed on subjects with preexisting disease; thus, the possibility that Cys might be marker of the disease itself, and not feature involved in its progression, cannot be ruled out. Conversely, in our series we observed a chronic disturbance of the cellular redox state, in terms of high total Cys levels, in the absence of overt vascular disease. Indeed, we recently showed, in otherwise healthy subjects, that Cys is a stronger marker of endothelial dysfunction, in terms of abnormal flow-mediated dilation, than clinical and all other biochemical variables explored in that study, including homocysteine levels at baseline and after methionine load [25].

Previous work on plasma thiol oxidation in healthy subjects [26, 27] described an association between total Cys and older age, male gender, body mass index (BMI), higher cholesterol, and diastolic blood pressure. Furthermore, higher levels of these risk factors were found to be associated with significant increases in total Cys with time. Our findings confirm that, in the presence of cardiovascular RF, a “thiol stress” is the major demonstrable alteration in plasma redox biochemistry, and its extent, in terms of Cys oxidation, is related to the total RF burden.

Many forms of thiol oxidation are reversible. These modifications, considered as “redox regulation” rather than “oxidative stress”, comprise mainly the formation of mixed disulphides between proteins and glutathione. It has been suggested that the increased vascular risk associated with elevated plasma tHcy may be partly explained by an associated fall in plasma total Cys [28]. However, other reports [8, 29] demonstrated that in healthy normohomocysteinaemic subjects, Cys tended to be more oxidized than glutathione. These authors suggested that, while the redox state of plasma glutathione is a response to both intracellular and extracellular oxidative processes, Cys redox state more accurately reflects the increased oxidative events in aging. Our findings in subjects without cardiovascular disease shed light on the prooxidant effect of Cys in conjunction with the RF burden.

The lack of association between plasma glutathione and RF burden might be ascribed to the higher proportion in group 2 and 3 of subjects treated by drug classes known to influence the antioxidant profile. Therefore, the susceptibility to disease progression should be ascribed mainly to increased oxidative processes, in terms of higher total plasma Cys levels, rather than to weakening of the antioxidant defence.

Free MDA is a well-known index of lipid peroxidation and marker of oxidative stress. In our study population, MDA levels showed an increasing trend (Table 2) in the unadjusted regression analysis, but no relationship between MDA and RF was evident after controlling for age and gender. Indeed, the smaller size and wider variations in MDA levels or the higher proportion of subjects on drug treatment in group 3 subjects might have mitigated the severity of oxidative stress, in terms of lipid peroxidation [30, 31].

Some limitations of our study should be considered. First, according to the known distribution of RF in the general population, an unbalance in age and gender was present in this series; to minimize its impact, we controlled for these factors in the statistical analysis.

We did not directly measure oxidized glutathione and Cys, so we could not reliably determine the redox state of the reduced/oxidized gluthatione and Cys pair, which were both recently found predictive of increased intima-media thickness in healthy subjects free from cardiovascular RFs [32]. However, to optimize preanalytical procedures for the assessment of redox markers in estimating cardiovascular risk, a more rapid collection and processing of samples, such as the one adopted for total and reduced rather than oxidized forms determination, are warranted.

We performed a separate analysis according to risk factor type, to elucidate whether specific risk factors were associated with a different redox pattern. Among redox variables, only MDA was associated with a specific risk factor, unsurprisingly hypercholesterolemia. However, the number of patients with a single risk factor in this series was low to derive definite conclusions from this finding.

Finally, the demonstrated association between RF burden and number of daily administered drugs appears obvious. We specifically decided to test this association because we wished to verify whether drugs with antioxidant properties would affect the redox state in different RF groups; total Cys remained independently associated to a higher RF burden when controlling for treatment. Furthermore, antioxidant drug treatment should have weakened, rather than increased, the difference in total Cys between RF groups.

In conclusion, our findings and the variable number of RFs support a prooxidant effect of Cys in conjunction with RFs in subjects without cardiovascular disease burden and shed light on the pathophysiologic role of redox state unbalance in preclinical atherosclerosis. The next step will be to verify the predictive capability of these biomarkers over a long follow-up period.

Since management of cardiovascular risk in asymptomatic individuals focuses on assessing traditional RFs, estimating risk, and modifying RFs through lifestyle changes or drug treatment, markers of possible mediators of the disease or noninvasive procedures that test for the likelihood of atherosclerotic burden are advisable. As pro-oxidant processes are involved in the initial phases of atherosclerosis, sensitive and cheap redox parameters appear helpful in risk assessment, to better identify asymptomatic subjects at high-intermediate risk. Those patients might benefit by therapeutic strategies able to provide the first line of defence during peroxyl radical attack; this concept should be the basis of intervention studies.

## Figures and Tables

**Table 1:** Clinical and biochemical characteristics according to risk factors burden.

	All patients (*n* = 304)	Group 0 (*n* = 81)	Group 1 (*n* = 95)	Group 2 (*n* = 80)	Group 3 (*n* = 48)
Age (years)	53 [43; 63]	44 [34; 53]	49 [39; 60]	59 [50; 67]	59 [53; 66]
Male gender (%)	169 (56%)	35 (43%)	51 (54%)	52 (65%)	31 (65%)
Body mass index (kg/m^2^)	25 [22; 28]	22 [21; 25]	25 [23; 27]	25 [23; 28]	28 [26; 31]
Fasting glucose (mg/dL)	93 [84; 109]	87 [80; 89]	89 [82; 96]	98 [87; 125]	120 [100; 147]
Total cholesterol (mg/dL)	200 [176; 230]	195 [171; 226]	197 [173; 216]	204 [179; 248]	217 [185; 244]
HDL cholesterol (mg/dL)	56 [47; 67]	63 [51; 73]	56 [50; 65]	50 [42; 62]	52 [46; 66]
LDL cholesterol (mg/dL)	117 [96; 146]	104 [84; 137]	118 [102; 141]	122 [98; 166]	115 [98; 164]
Triglycerides (mg/dL)	106 [71; 145]	85 [55; 127]	92 [61; 124]	127 [91; 174]	129 [96; 212]
Creatinine (mg/dL)	0.83 [0.70; 0.98]	0.83 [0.72; 0.99]	0.79 [0.68; 0.97]	0.84 [0.70; 0.99]	0.89 [0.72; 1.01]
Serum folate (ng/mL)	6.1 [4.6; 8.1]	6.2 [4.8; 8.8]	6.1 [4.5; 7.8]	5.3 [4.1; 7.7]	7.5 [5.5; 9.9]
Vitamin B_12_ (pg/mL)	396 [284; 530]	465 [367; 588]	362 [275; 450]	386 [265; 532]	365 [230; 501]

Data are expressed as median value and interquartile range [I; III] or number of patients (percentage).

**Table 2:** Redox characteristics according to risk factors burden.

	All patients (*n* = 304)	Group 0 (*n* = 81)	Group 1 (*n* = 95)	Group 2 (*n* = 80)	Group 3 (*n* = 48)
Plasma total thiols (*μ*m/L)					
Homocysteine	9.5 [7.4; 14.1]	9.0 [7.1; 10.9]	9.2 [7.4; 13.8]	11.5 [7.8; 18.6]	9.9 [7.5; 19.0]
Cysteine	250 [210; 291]	228 [197; 269]	253 [203; 280]	260 [223; 305]	280 [230; 320]
Cysteinylglycine	30.8 [23.9; 41.9]	32.7 [24.7; 43.4]	30.2 [24.0; 44.0]	31.1 [23.7; 37.6]	29.9 [22.4; 37.3]
Glutathione	5.5 [4.1; 7.3]	5.9 [4.3; 8.0]	5.6 [4.0; 7.0]	5.4 [4.2; 7.3]	5.5 [3.7; 0.7.5]
Plasma reduced thiols (*μ*m/L)					
Homocysteine	0.16 [0.10; 0.24]	0.13 [0.10; 0.21]	0.16 [0.10; 0.24]	0.19 [0.11; 0.29]	0.14 [0.07; 0.28]
Cysteine	7.9 [5.7; 9.4]	7.9 [6.2; 9.0]	7.7 [5.7; 9.1]	7.9 [5.7; 10.0]	8.1 [5.7; 10.1]
Cysteinylglycine	3.3 [2.1; 4.5]	3.5 [2.2; 5.3]	3.5 [2.1; 4.9]	3.3 [2.2; 4.3]	2.9 [1.4; 3.7]
Glutathione	1.9 [1.3; 3.8]	1.9 [1.3; 3.9]	1.8 [1.1; 3.5]	2.1 [1.4; 4.2]	2.1 [1.3; 4.4]
Free malondialdehyde (*μ*mol/L)	0.76 [0.47; 1.35]	0.62 [0.36; 0.89]	0.70 [0.45; 1.30]	0.94 [0.68; 1.33]	1.26 [0.65; 1.49]
Blood reduced glutathione (*μ*mol/L)	655 [496; 824]	655 [451; 821]	705 [536; 859]	663 [454; 842]	569 [499; 771]

Data are expressed as median value and interquartile range [I; III].

**Table 3:** Current medications in risk factor groups.

	Total (*n* = 223)	Group 1 (*n* = 95)	Group 2 (*n* = 80)	Group 3 (*n* = 48)
ACE inhibitors	43 (19%)	3 (3%)	20 (25%)	20 (42%)
Angiotensin II receptor antagonists	18 (8%)	4 (4%)	6 (7%)	8 (17%)
*beta*-blockers	27 (12%)	2 (2%)	14 (17%)	11 (23%)
Diuretics	11 (5%)	3 (3%)	3 (4%)	5 (10%)
Calcium-channel blockers	36 (16%)	3 (3%)	19 (24%)	14 (29%)
Antiplatelet agents	28 (12%)	6 (6%)	13 (16%)	9 (19%)
Statins	33 (15%)	3 (3%)	12 (15%)	18 (38%)
Antidiabetic agents	42 (19%)	7 (7%)	16 (20%)	19 (40%)
Number of drugs*	1 [0; 2]	0 [0; 1]	1 [0; 2]	2 [1; 3]

Data are expressed as number of patients (percentage).

*Number of drugs: sum of all drug classes reported above in the table, presented as median and interquartile range [I; III].

ACE: angiotensin converting enzyme.

**Table 4:** Age and gender-adjusted ordinal logistic regression analysis versus number of risk factors.

	*P* value	Adjusted OR	95% CI
Creatinine (mg/mL)	0.14	0.384	0.107–1.377
Serum folate (ng/mL)	0.96	1.002	0.930–1.079
Vitamin B_12_ (pg/mL)	0.06	0.999	0.998–1.011
Plasma total thiols (*μ*m/L)			
Cysteine	0.03	1.004	1.002–1.008
Cysteinylglycine	0.28	1.007	0.995–1.020
Glutathione	0.21	1.052	0.970–1.140
Plasma reduced thiols (*μ*m/L)			
Cysteine	0.77	1.009	0.951–1.070
Cysteinylglycine	0.60	0.977	0.894–1.067
Glutathione	0.08	1.064	0.992–1.142
Free malondialdehyde (*μ*mol/L)	0.12	1.438	0.905–2.283
Blood reduced glutathione (*μ*mol/L)	0.86	1.000	0.998–1.002
Number of drugs	<0.001	2.983	2.303–3.864

OR, odds ratio; CI, confidence interval.
